# Differences in seeking breast cancer care between screening eligible versus ineligible Palestinian women

**DOI:** 10.1038/s41598-025-10605-x

**Published:** 2025-07-09

**Authors:** Mohamedraed Elshami, Maram Albandak, Mohammed Alser, Ibrahim Al-Slaibi, Faten Darwish Usrof, Malak Ayman Mousa Qawasmi, Islam Osama Ismail, Roba Jamal Ghithan, Heba Mahmoud Okshiya, Nouran Ramzi Shaban Shurrab, Ibtisam Ismail Mahfouz, Aseel AbdulQader Fannon, Mona Radi Mohammad Hawa, Narmeen Giacaman, Manar Ahmaro, Rula Khader Zaatreh, Wafa Aqel AbuKhalil, Noor Khairi Melhim, Ruba Jamal Madbouh, Hala Jamal Abu Hziema, Raghad Abed-Allateef Lahlooh, Sara Nawaf Ubaiat, Nour Ali Jaffal, Reem Khaled Alawna, Salsabeel Naeem Abed, Bessan Nimer Ali Abuzahra, Aya Jawad Abu Kwaik, Mays Hafez Dodin, Raghad Othman Taha, Dina Mohammed Alashqar, Roaa Abd-al-Fattah Mubarak, Tasneem Smerat, Shurouq I. Albarqi, Mysoon Abu-El-Noor, Nasser Abu-El-Noor, Bettina Bottcher

**Affiliations:** 1 Division of Surgical Oncology, Department of Surgery, University Hospitals Cleveland Medical Center, 11100 Euclid Avenue, Cleveland, OH 44106 USA; 2 Ministry of Health, Gaza, Palestine; 3 Department of Internal Medicine, University of Toledo, Toledo, OH USA; 4 Faculty of Medicine, Al-Quds University, Jerusalem, Palestine; 5 United Nations Relief and Works Agency for Palestine Refugees (UNRWA), Gaza, Palestine; 6 Almakassed Hospital, Jerusalem, Palestine; 7 Department of a Medical Laboratory Sciences, Faculty of Health Sciences, Islamic University of Gaza, Gaza City, Palestine; 8 Palestinian Red Crescent Hospital-Halhoul, Hebron, Palestine; 9 Al-Shifa Hospital, Gaza, Palestine; 10 Faculty of Medicine, Islamic University of Gaza, Gaza, Palestine; 11 Tulkarem Governmental Hospital, Tulkarem, Palestine; 12 Clalit Al-Makassed Medical Center, Jerusalem, Palestine; 13 Department of Pharmacy, An-Najah National University, Nablus, Palestine; 14 Department of Internal Medicine, An-Najah National University Hospital, Nablus, Palestine; 15 Faculty of Dentistry, Al-Quds University, Jerusalem, Palestine; 16 Faculty of Medicine and Health Sciences, Palestine Polytechnic University, Hebron, Palestine; 17 Faculty of Pharmacy, Al-Azhar University of Gaza, Gaza, Palestine; 18 Faculty of Nursing, Islamic University of Gaza, Gaza, Palestine

**Keywords:** Breast cancer, Early presentation, Early diagnosis, Symptoms, Health education, Palestine, Breast cancer, Cancer screening

## Abstract

In Palestine, breast cancer (BC) is the most prevalent cancer among women. This study explored the relationship between eligibility for BC screening and the time taken for seeking medical advice for possible BC symptoms. It also identified the barriers hindering early presentation among screening-eligible and ineligible Palestinian women. Palestinian females from governmental hospitals, primary healthcare centers, and diverse public venues across 11 governorates within Palestine were recruited using convenience sampling. A modified, translated-into-Arabic version of the BC Awareness Measure was utilized for data collection. The questionnaire comprised three sections: sociodemographic information, duration for seeking medical advice upon recognizing each of 13 possible BC symptoms, and barriers impeding early presentation. The final analysis included 2796 participants, with 2168 (77.5%) ineligible for BC screening and 628 (22.5%) eligible. The proportion of participants who would seek medical advice immediately varied depending on the nature of BC symptoms. Approximately half of participants in both the screening-eligible (n = 1097, 50.6%) and ineligible (n = 293, 46.7%) groups indicated that they would seek immediate medical advice for ‘lump or thickening in the breast.’ Similarly, about half of participants in both groups reported an inclination to seek immediate medical advice for ‘nipple discharge or bleeding’ (screening-eligible: n = 954, 44.0%; screening-ineligible: n = 280, 44.6%). Less than half of participants would seek immediate medical advice for other BC symptoms. Screening-eligible women were more likely to seek advice within a week for six out of 13 possible BC symptoms than screening-ineligible women. Among screening-eligible participants, 9.9% (n = 62) acknowledged a delay in seeking medical attention upon recognizing a potential BC symptom. Emotional barriers were most frequently reported, with ‘disliking the visit to healthcare facilities’ identified as the leading barrier among screening-eligible participants (n = 38, 61.3%). No association was found between BC screening eligibility and the total or type-specific number of reported barriers. Differences in immediate medical advice seeking for various BC symptoms were observed between screening-eligible and ineligible groups. Emotional barriers were the most prevalent in both groups. Tailored interventions to enhance BC awareness and address barriers to early presentation are essential.

## Introduction

Breast cancer (BC) has emerged as the most frequently diagnosed cancer globally, presenting a significant challenge to public health^1^. Currently, BC constitutes 1 in 8 newly diagnosed cancer cases, amounting to 2.3 million new cases across both genders^1^. Projections indicate a substantial increase, with an expected 40% rise in newly diagnosed BC cases, reaching approximately 3 million annually by 2040. In addition, BC-related deaths are projected to surge by over 50%, escalating from 685,000 in 2020 to 1 million in 2040^2^. Notably, BC is the most prevalent cancer among Palestinian women, accounting for 35.6% of documented cases of female cancers in Palestine. The age-standardized incidence and mortality rates are 53.5 and 22.6 per 100,000 female population, respectively, in Palestine^3^.

Early detection of BC significantly increases the likelihood of successful treatment compared to later-stage diagnoses^4^. In countries with low-resource settings, many BC patients face delays in diagnosis, leading to advanced-stage detection and reduced survival rates^5,6^. In Palestine specifically, advanced stages account for over 60% of BC cases, reducing the likelihood of positive outcomes^7^. Furthermore, despite the availability of a fully accessible and cost-free screening program^8^, the utilization of mammography in Palestine continues to be notably low^9^. This underscores the urgency of emphasizing timely detection of BC through screening measures and improved awareness of BC, as these factors play a crucial role in influencing prognosis and reducing the morbidity and mortality rates associated with BC^10^.

Nevertheless, various barriers, including emotional, service-related, and practical barriers, may hinder women from promptly seeking medical attention^11^. Assessing the current understanding of BC and barriers associated with timely presentation among Palestinian women eligible for BC screening is imperative, especially given the documented low participation rates in BC screening^12^. Therefore, this national study aimed to examine the association between BC screening eligibility and the anticipated time for seeking medical advice among Palestinian women. It also aimed to identify and compare barriers to early presentation between screening-eligible and ineligible women.

## Methods

### Study design and population

This nationwide cross-sectional study was carried out from July 2019 to March 2020, targeting adult Palestinian women living in Jerusalem, the West Bank, and the Gaza Strip. Exclusion criteria included women visiting oncology departments or clinics during the data collection period, women with history of any breast disease(s), those with healthcare background, non-Palestinian nationals, women with family history of cancer, women who underwent screening mammography, and those unable to complete the questionnaire.

### Sampling methods

Eligible women were enrolled utilizing convenience sampling from specified data collection sites, including primary healthcare centers, governmental hospitals, and public venues distributed across 11 governorates in Palestine (seven in the West Bank and Jerusalem, and four in the Gaza Strip). Public venues included restaurants, markets, downtown areas, parks, malls, churches, mosques, and public transportation stations. The inclusion of participants from various locations and governorates across Palestine was intended to enhance the representativeness and diversity of the study cohort^13–15^.

### Questionnaire and data collection

A modified version of the validated Breast Cancer Awareness Measure (BCAM) assessment tool was utilized for data collection^16^. The original BCAM in English underwent translation into Arabic by two bilingual experts, followed by back-translation into English by two different bilingual experts. Evaluation of the Arabic BCAM version involved five experts specializing in BC, public health, and survey design to ensure content validity and translation accuracy. A pilot study with 35 participants was conducted to assess the clarity of questions in the Arabic BCAM. Responses from the pilot study were excluded from the final analysis. The internal consistency of the Arabic BCAM was assessed using Cronbach’s Alpha, which yielded a satisfactory value of 0.75.

The study questionnaire consisted of three sections. The first section gathered sociodemographic information including age, marital status, highest level of education, employment status, monthly income, place of residency, presence of chronic diseases, knowing someone diagnosed with cancer, and the site of data collection. The second section evaluated the anticipated time women would take to seek medical consultation for each of 13 possible BC symptoms. Eleven symptoms were retained from the original BCAM^16^, with the addition of two other symptoms, namely ‘extreme generalized fatigue’ and ‘unexplained weight loss’ based on previous studies^11,17,18^. The third section explored perceived barriers to early presentation, categorized into emotional, service-related, and practical barriers.

The data collection process utilized Kobo Toolbox, a secure and user-friendly platform accessible via smartphones^19^. Trained female data collectors with a medical background underwent comprehensive training to conduct face-to-face interviews for questionnaire completion through the Kobo Toolbox platform. The inclusion of female data collectors aimed to mitigate potential discomfort among women when responding to sensitive questions.

### Exposure and outcome

Our group recently demonstrated various proportions of participants who would immediately seek medical advice for possible BC symptoms and identified different barriers to early presentation within the general population^20^. However, there exists a gap in the literature regarding the specific examination of these aspects among women who are eligible for BC screening compared to those who are not. Subsequently, the primary exposure in this study was the eligibility to undergo BC screening, defined as aging 40 to 74 years while having an average-risk of developing BC (i.e., no family history of cancer or personal history of breast disease). The primary outcome measure was the proportion of participants who would seek immediate medical advice for each BC symptom, stratified by eligibility to undergo BC screening. The secondary outcome measure was the proportion of reported barriers that could potentially hinder a visit to the doctor when a BC symptom is recognized.

### Statistical analysis

In Palestine, BC screening is recommended for women aged 40 and above^21^. Accordingly, age was categorized into two groups: 18–39 years and ≥ 40 years. Similarly, monthly income was divided into two categories: < 1450 NIS and ≥ 1450 NIS, based on the minimum wage in Palestine, approximately equivalent to $450^22^.

The median and interquartile range (IQR) were utilized to depict continuous variables exhibiting non-normal distributions. Conversely, categorical variables were summarized using frequencies and percentages. Baseline characteristics between screening-eligible and ineligible participants were compared using the Kruskal–Wallis test for continuous variables and the Pearson chi-square test for categorical variables.

Symptoms of BC were classified into three categories: (i) breast symptoms, (ii) nipple symptoms, and (iii) other symptoms. Additionally, the expected timeframe to seek medical advice for potential BC symptoms was divided into four groups: immediate (≤ 24 h), ≤ 7 days, > 7 days, and never. The frequency and percentage distribution of anticipated timeframes were analyzed, and a comparison between screening-eligible and ineligible participants was conducted using the Pearson chi-square test.

Multivariable logistic regression analysis was employed to assess the association between eligibility for BC screening and the expected time to seek medical advice for potential BC symptoms. The multivariable model was adjusted for covariates including age group, educational level, employment status, monthly income, marital status, place of residence, presence of a chronic disease, familiarity with someone diagnosed with cancer, and site of data collection. This model was determined a priori based on prior studies^13–15,20^.

In line with prior studies^16,20,21^, barriers to early presentation were categorized into emotional, service-related, and practical barriers. The frequencies and percentages of reported barriers were analyzed and compared between screening-eligible and ineligible participants using the Pearson chi-square test. The median number of barriers in each category served as the cutoff point to dichotomize the number of displayed barriers (overall, emotional, service, and practical). Multivariable logistic regression analysis was then conducted to explore the association between BC screening eligibility and the presence of at least the median number of barriers to early presentation, utilizing the previously mentioned multivariable model.

Missing data were hypothesized to be missed completely at random and were handled through a complete case analysis approach. The data analysis was conducted using Stata software version 17.0 (StataCorp, College Station, TX).

## Results

### Participant characteristics

The final analysis included 2796 participants, with 2168 (77.5%) ineligible for BC screening and 628 (22.5%) eligible (Table 1). Participants in the BC screening-eligible group were older, exhibited higher unemployment rates, reported lower monthly income and educational level, and suffered from more frequent chronic diseases.

**Table 1:** Characteristics of study participants.

Characteristic	Screening-ineligible (n = 2168)	Screening-eligible (n = 628)	p-value
Age, median [IQR]	26.0 [22.0, 31.0]	48.0 [43.0, 55.0]	< 0.001
Educational level, n (%)			
Secondary or below	1060 (48.9)	521 (83.0)	< 0.001
Post-secondary	1108 (51.1)	107 (17.0)	
Occupation, n (%)			
Unemployed/housewife	1363 (62.9)	518 (82.5)	< 0.001
Employed	405 (18.7)	109 (17.4)	
Student	400 (18.5)	1 (0.2)	
Monthly income ≥ 1450 NIS, n (%)	1232 (56.8)	317 (50.5)	0.005
Marital status, n (%)			
Single	782 (36.1)	30 (4.8)	< 0.001
Married	1338 (61.7)	508 (80.9)	
Divorced/widowed	48 (2.2)	90 (14.3)	
Residency, n (%)			
Gaza Strip	1161 (53.6)	290 (46.2)	< 0.001
West Bank and Jerusalem	1007 (46.4)	338 (53.8)	
Having a chronic disease, n (%)	141 (6.5)	272 (43.3)	< 0.001
Knowing someone with cancer, n (%)	311 (14.3)	130 (20.7)	< 0.001
Site of data collection, n (%)			
Public spaces	814 (37.5)	150 (23.9)	< 0.001
Hospitals	722 (33.3)	354 (56.4)	
Primary healthcare centers	632 (29.2)	124 (19.7)	

*n* number of participants, *IQR* interquartile range.

### Immediate seeking of medical advice for a possible BC symptom in BC screening-eligible vs ineligible groups

Approximately half of participants in both the screening-eligible (n = 1097, 50.6%) and ineligible (n = 293, 46.7%) groups indicated that they would seek immediate medical advice for ‘lump or thickening in the breast’ (Table 2). However, less than one third of participants in both groups would seek medical advice immediately for other breast symptoms.

**Table 2:** Summary of the reported time to seek medical advice for a possible breast cancer symptom, stratified by eligibility status to undergo a screening mammography.

Category of sym ptoms	BC sign/symptom	Screening-ineligible (n = 2168)	Screening-eligible (n = 628)	p-value
Breast symptoms	A lump or thickening in the breast			
	Immediately	1097 (50.6)	293 (46.7)	0.13
	≤ 7 days	970 (44.7)	294 (46.8)	
	> 7 days	45 (2.1)	20 (3.2)	
	Never	56 (2.6)	21 (3.3)	
	Puckering or dimpling of the breast skin			
	Immediately	575 (26.5)	203 (32.3)	0.018
	≤ 7 days	1011 (46.6)	285 (45.4)	
	> 7 days	56 (2.6)	15 (2.4)	
	Never	526 (24.3)	125 (19.9)	
	Pain in one of the breasts or armpits			
	Immediately	549 (25.3)	186 (29.6)	0.046
	≤ 7 days	1099 (50.7)	307 (48.9)	
	> 7 days	113 (5.2)	20 (3.2)	
	Never	407 (18.8)	115 (18.3)	
	Redness of the breast skin			
	Immediately	473 (21.8)	182 (29.0)	< 0.001
	≤ 7 days	944 (43.5)	295 (47.0)	
	> 7 days	74 (3.4)	10 (1.6)	
	Never	677 (31.2)	141 (22.5)	
Nipple symptoms	Discharge or bleeding from the nipple			
	Immediately	954 (44.0)	280 (44.6)	0.33
	≤ 7 days	1068 (49.3)	298 (47.5)	
	> 7 days	37 (1.7)	8 (1.3)	
	Never	109 (5.0)	42 (6.7)	
	Pulling in of the nipple			
	Immediately	607 (28.0)	214 (34.1)	0.028
	≤ 7 days	1024 (47.2)	276 (43.9)	
	> 7 days	72 (3.3)	16 (2.5)	
	Never	465 (21.4)	122 (19.4)	
	Change in the position of the nipple			
	Immediately	585 (27.0)	212 (33.8)	0.010
	≤ 7 days	1074 (49.5)	289 (46.0)	
	> 7 days	69 (3.2)	16 (2.5)	
	Never	440 (20.3)	111 (17.7)	
	Nipple rash			
	Immediately	572 (26.4)	195 (31.1)	0.040
	≤ 7 days	1036 (47.8)	299 (47.6)	
	> 7 days	53 (2.4)	10 (1.6)	
	Never	507 (23.4)	124 (19.7)	
Other symptoms	Lump or thickening under the armpit			
	Immediately	937 (43.2)	278 (44.3)	0.90
	≤ 7 days	1020 (47.0)	288 (45.9)	
	> 7 days	54 (2.5)	18 (2.9)	
	Never	157 (7.2)	44 (7.0)	
	Changes in the shape of the breast or nipple			
	Immediately	671 (31.0)	235 (37.4)	0.011
	≤ 7 days	1037 (47.8)	286 (45.5)	
	> 7 days	65 (3.0)	17 (2.7)	
	Never	395 (18.2)	90 (14.3)	
	Changes in the size of the breast or nipple			
	Immediately	628 (29.0)	229 (36.5)	< 0.001
	≤ 7 days	999 (46.1)	282 (44.9)	
	> 7 days	70 (3.2)	15 (2.4)	
	Never	471 (21.7)	102 (16.2)	
	Unexplained weight loss			
	Immediately	283 (13.1)	114 (18.2)	< 0.001
	≤ 7 days	624 (28.8)	210 (33.4)	
	> 7 days	242 (11.2)	70 (11.1)	
	Never	1019 (47.0)	234 (37.3)	
	Extreme generalized fatigue			
	Immediately	241 (11.1)	88 (14.0)	0.006
	≤ 7 days	832 (38.4)	265 (42.2)	
	> 7 days	84 (3.9)	30 (4.8)	
	Never	1011 (46.6)	245 (39.0)	

*n* number of participants.

Similarly, about half of participants in both groups reported an inclination to seek immediate medical advice for ‘nipple discharge or bleeding’ (screening-eligible: n = 954, 44.0%; screening-ineligible: n = 280, 44.6%). About one third of study participants or less reported immediate seeking of medical advice for other nipple symptoms.

Less than half of participants would seek immediate medical advice for other BC symptoms, with ‘lump or thickening under the armpit’ reported as the most concerning symptom in both groups (screening-eligible: n = 937, 43.2%; screening-ineligible: n = 278, 44.3%). The proportion of immediate seeking of medical advice was lower for other BC symptoms, with ‘extreme generalized fatigue’ receiving the least priority for immediate medical attention in both groups (screening-eligible: n = 241, 11.1%; screening-ineligible: n = 88, 14.0%).

### Association between eligibility for BC screening and no seeking of medical advice

Screening-eligible women had a lower likelihood of not seeking medical advice for six out of 13 possible BC symptoms, namely ‘redness of the breast skin’ (OR = 0.68, 95% CI 0.53–0.88), ‘nipple rash’ (OR = 0.76, 95% CI 0.58–0.99), ‘changes in the shape of the breast or nipple’ (OR = 0.72, 95% CI 0.53–0.97), ‘changes in the size of the breast or nipple’ (OR = 0.69, 95% CI 0.52–0.92), ‘unexplained weight loss’ (OR = 0.65, 95% CI 0.52–0.81), and ‘extreme generalized fatigue’ (OR = 0.70, 95% CI 0.57–0.87) (Table 3). However, screening-eligible women had a higher likelihood of not seeking medical advice for ‘discharge or bleeding from the nipple’ (OR = 1.63, 95% CI 1.04–2.56) compared to their screening-ineligible counterparts.

**Table 3:** Multivariable logistic regression analyzing the association between no seeking of medical advice and being eligible to undergo a screening mammography.

Eligibility to undergo a screening mammography	A) Breast symptoms									
	A lump or thickening in the breast		Pain in one of the breasts or armpits		Puckering or dimpling of the breast skin		Redness of the breast skin			
	AOR (95% CI)	p-value	AOR (95% CI)	p-value	AOR (95% CI)	p-value	AOR (95% CI)	p-value		
No	Ref	Ref	Ref	Ref	Ref	Ref	Ref	Ref		
Yes	1.49 (0.79- 2.83)	0.22	1.06 (0.80- 1.40)	0.70	0.77 (0.59- 1.00)	0.051	0.68 (0.53- 0.88)	0.003		
Eligibility to undergo a screening mammography	B) Nipple symptoms									
	Discharge or bleeding from the nipple		Nipple rash		Change in the position of the nipple		Pulling in of the nipple			
	AOR (95% CI)	p-value	AOR (95% CI)	p-value	AOR (95% CI)	p-value	AOR (95% CI)	p-value		
No	Ref	Ref	Ref	Ref	Ref	Ref	Ref	Ref		
Yes	1.63 (1.04- 2.56)	0.032	0.76 (0.58- 0.99)	0.042	0.97 (0.73- 1.28)	0.81	0.92 (0.70- 1.21)	0.56		
Eligibility to undergo a screening mammography	C) Other symptoms									
	Lump or thickening under the armpit		Changes in the shape of the breast or nipple		Changes in the size of the breast or nipple		Unexplained weight loss		Extreme generalized fatigue	
	AOR (95% CI)	p-value	AOR (95% CI)	p-value	AOR (95% CI)	p-value	AOR (95% CI)	p-value	AOR (95% CI)	p-value
No	Ref	Ref	Ref	Ref	Ref	Ref	Ref	Ref	Ref	Ref
Yes	1.13 (0.74- 1.74)	0.56	0.72 (0.53- 0.97)	0.032	0.69 (0.52- 0.92)	0.011	0.65 (0.52- 0.81)	< 0.001	0.70 (0.57- 0.87)	0.001

*AOR* adjusted odds ratio, *CI* confidence interval.

All analyses were adjusted for educational level, occupation, monthly income, marital status, residency, having a chronic disease, knowing someone with cancer, and site of data collection.

### Association between eligibility for BC screening and seeking medical advice within a week

Screening-eligible women had a higher likelihood of seeking medical advice within a week for six out of 13 possible BC symptoms, namely ‘redness of the breast skin’ (OR = 1.59, 95% CI 1.24–2.03), ‘nipple rash’ (OR = 1.38, 95% CI 1.06–1.79), ‘changes in the shape of the breast or nipple’ (OR = 1.36, 95% CI 1.03–1.81), ‘changes in the size of the breast or nipple’ (OR = 1.44, 95% CI 1.10–1.88), ‘unexplained weight loss’ (OR = 1.42, 95% CI 1.14–1.75), and ‘extreme generalized fatigue’ (OR = 1.41, 95% CI 1.14–1.75) compared to their screening ineligible counterparts (Table 4).

**Table 4:** Multivariable logistic regression analyzing the association between seeking medical advice within a week and being eligible to undergo a screening mammography.

Eligibility to undergo a screening mammography	A) Breast symptoms									
	A lump or thickening in the breast		Pain in one of the breasts or armpits		Puckering or dimpling of the breast skin		Redness of the breast skin			
	AOR (95% CI)	p-value	AOR (95% CI)	p-value	AOR (95% CI)	p-value	AOR (95% CI)	p-value		
No	Ref	Ref	Ref	Ref	Ref	Ref	Ref	Ref		
Yes	0.80 (0.49- 1.29)	0.35	1.08 (0.83- 1.40)	0.57	1.25 (0.97- 1.62)	0.09	1.59 (1.24- 2.03)	< 0.001		
Eligibility to undergo a screening mammography	B) Nipple symptoms									
	Discharge or bleeding from the nipple		Nipple rash		Change in the position of the nipple		Pulling in of the nipple			
	AOR (95% CI)	p-value	AOR (95% CI)	p-value	AOR (95% CI)	p-value	AOR (95% CI)	p-value		
No	Ref	Ref	Ref	Ref	Ref	Ref	Ref	Ref		
Yes	0.73 (0.48- 1.10)	0.13	1.38 (1.06- 1.79)	0.017	1.09 (0.84- 1.42)	0.51	1.15 (0.89- 1.49)	0.29		
Eligibility to undergo a screening mammography	C) Other symptoms									
	Lump or thickening under the armpit		Changes in the shape of the breast or nipple		Changes in the size of the breast or nipple		Unexplained weight loss		Extreme generalized fatigue	
	AOR (95% CI)	p-value	AOR (95% CI)	p-value	AOR (95% CI)	p-value	AOR (95% CI)	p-value	AOR (95% CI)	p-value
No	Ref	Ref	Ref	Ref	Ref	Ref	Ref	Ref	Ref	Ref
Yes	0.89 (0.61- 1.28)	0.52	1.36 (1.03- 1.81)	0.031	1.44 (1.10- 1.88)	0.008	1.42 (1.14- 1.75)	0.002	1.41 (1.14- 1.75)	0.002

*AOR* adjusted odds ratio, *CI* confidence interval.

All analyses were adjusted for educational level, occupation, monthly income, marital status, residency, having a chronic disease, knowing someone with cancer, and site of data collection.

### Barriers to early presentation and eligibility for BC screening

Among screening-eligible participants, 9.9% (n = 62) acknowledged a delay in seeking medical attention upon recognizing a potential BC symptom, reporting at least one barrier to early presentation (Table 5). The most prevalent barriers to seeking medical advice for potential BC symptoms were emotional in nature, with ‘disliking the visit to healthcare facilities’ identified as the most reported barrier among screening-eligible participants (n = 38, 61.3%) and ‘feeling worried about what the doctor might find’ in the screening-ineligible group (n = 133, 57.6%). Regarding service barriers, ‘the small number of female doctors in the Palestinian Ministry of Health facilities’ was the most reported barrier in both the screening-eligible (n = 19, 30.6%) and ineligible (n = 77, 33.3%) groups. The most commonly reported practical barrier in the screening-eligible group was ‘having other things to do’ (n = 35, 56.5%), while it was ‘would think that the symptom is not something important to see the doctor for’ (n = 109, 47.2%) in the screening-ineligible group.

**Table 5:** Summary of the reported barriers to early presentation, stratified by eligibility status to undergo a screening mammography.

Category	Barrier	Screening- ineligible (n = 231)	Screening-eligible (n = 62)	OR* (95% CI)	p-value
Emotional	Feeling worried about what a doctor might find	133 (57.6)	35 (56.5)	0.90 (0.44- 1.82)	0.76
	Feeling scared	130 (56.3)	36 (58.1)	0.96 (0.47- 1.97)	0.92
	Would not feel comfortable to be examined by a male doctor	112 (48.5)	31 (50.0)	0.82 (0.40- 1.68)	0.60
	Disliking the visit to healthcare facilities	110 (47.6)	38 (61.3)	1.33 (0.65- 2.72)	0.44
	Would prefer not to know the reason of the symptom	87 (37.7)	32 (51.6)	1.06 (0.52- 2.20)	0.87
	Embarrassment	87 (37.7)	21 (33.9)	0.81 (0.38- 1.71)	0.58
	God is the ultimate healer so there in no need to go to the doctor	79 (34.2)	37 (59.7)	1.36 (0.66- 2.83)	0.40
	Would not trust the doctor	59 (25.5)	20 (32.3)	1.14 (0.51- 2.58)	0.75
	Would not feel confident talking about symptom with	52 (22.5)	8 (12.9)	0.47 (0.18- 1.25)	0.13
Service	The small number of female doctors in the Palestinian Ministry of Health facilities	77 (33.3)	19 (30.6)	1.06 (0.49- 2.26)	0.89
	Difficulty making an appointment with the doctor	68 (29.4)	19 (30.6)	0.57 (0.25- 1.31)	0.19
	Feeling worried about wasting doctor’s time	48 (20.8)	7 (11.3)	0.29 (0.10- 0.89)	0.031
	Difficulty talking to doctor	32 (13.9)	9 (14.5)	0.49 (0.17- 1.39)	0.18
Practical	Would think that symptom is not something important to see the doctor for	109 (47.2)	34 (54.8)	0.84 (0.41- 1.73)	0.63
	Too busy to go to the doctor	93 (40.3)	29 (46.8)	0.73 (0.35- 1.50)	0.39
	Has other things to do	93 (40.3)	35 (56.5)	0.97 (0.47- 2.03)	0.95
	Would try some herbs first	80 (34.6)	28 (45.2)	1.06 (0.51- 2.23)	0.87
	Financial hardship that will not allow seeing the doctor	64 (27.7)	21 (33.9)	0.55 (0.24- 1.28)	0.17
	Difficulty arranging transports to the doctor	42 (18.2)	16 (25.8)	0.90 (0.37- 2.22)	0.83
	Family or husband would refuse their wife being examined by a male doctor	52 (22.5)	10 (16.1)	0.42 (0.17- 1.04)	0.06
	Family or husband would refuse the visit to the doctor	18 (7.8)	4 (6.5)	0.66 (0.17- 2.54)	0.55

*n* number of participants, *OR* odds ratio, *CI* confidence interval.

*Adjusted for educational level, occupation, monthly income, marital status, residency, having a chronic disease, knowing someone with cancer, and site of data collection.

There was no association between eligibility for BC screening and the total number of reported barriers (Table 6). Similarly, no associations were found between BC screening eligibility and the reported number of each type of barriers, whether emotional, service-related, or practical.

**Table 6:** Multivariable logistic regression analyzing the association between having at least the median number of barriers to seek medical advice for a possible breast cancer symptom and being eligible to undergo a screening mammography.

Eligibility to undergo a screening mammography	All barriers		Emotional barriers		Service barriers		Practical barriers	
	AOR (95% CI)*	p-value	AOR (95% CI)*	p-value	AOR (95% CI)*	p-value	AOR (95% CI)*	p-value
No	Ref	Ref	Ref	Ref	Ref	Ref	Ref	Ref
Yes	0.74 (0.35- 1.56)	0.43	0.91 (0.44- 1.87)	0.79	0.54 (0.26- 1.12)	0.10	0.84 (0.38- 1.84)	0.66

*COR* crude odds ratio, *AOR* adjusted odds ratio, *CI* confidence interval.

*Adjusted for educational level, occupation, monthly income, marital status, residency, having a chronic disease, knowing someone with cancer, and site of data collection.

## Discussion

This study assessed the differences in early presentation and barriers to seeking medical advice between screening-eligible and ineligible women. The results indicate that the proportion of participants who would seek medical advice immediately varied for different BC symptoms among both groups. Emotional barriers were the most frequently reported barriers by study participants in both groups. Notably, there was no association found between eligibility for BC screening and the total number of reported barriers. Similarly, no associations were identified between BC screening eligibility and the reported number of each type of barriers, whether emotional, service-related, or practical.

Approximately half of participants in both the screening-eligible and ineligible groups expressed their intention to seek immediate medical advice for ‘lump or thickening in the breast’ and ‘nipple discharge or bleeding.’ For other BC symptoms, less than half of the participants in both groups expressed the intent for an immediate medical consultation, with ‘lump or thickening under the armpit’ identified as the most concerning symptom in both groups. This aligns with previous studies indicating that women exhibit a greater likelihood of promptly seeking medical assistance when experiencing symptoms related to a breast lump or thickening in the axilla^20,23,24^. Understanding the factors contributing to delays in seeking medical attention provides insights for tailored interventions, such as emphasizing other non-lump related BC symptoms in awareness campaigns. This can help reduce time intervals, leading to earlier diagnoses and lower mortality rates from BC in Palestine.

Interestingly, a study revealed that women with both lump and non-lump symptoms exhibited prolonged patient intervals compared to those with breast lump-only symptoms^25^. This suggests an increased tendency among women to normalize a breast lump when accompanied by additional non-lump-related breast symptoms^26^. It also underscores the significance of educational interventions tailored to expedite the diagnostic pathway for women presenting with non-lump symptoms. Improving the clarity of referral pathways, enhancing diagnostic services, and optimizing the limited human and infrastructure resources may contribute to more consistent and coordinated care. These measures have the potential to enhance accurate diagnoses at earlier stages and ultimately improving overall BC outcomes, especially in resource-limited countries like Palestine^27^.

This study revealed that screening-eligible women had a higher likelihood of promptly seeking medical advice within a week for six out of 13 possible BC symptoms. This could be attributed to better health literacy stemming from more frequent interactions with the healthcare system and the proactive health-seeking behavior encouraged by their healthcare providers^28^. Being aware of their eligibility for BC screening may have contributed to a greater sensitivity to potential symptoms, prompting them to seek medical advice promptly. Moreover, considering the younger age of screening-ineligible participants, it is plausible that they perceived their risk of BC to be lower and were less likely to attribute their symptoms to BC. Instead, they might have thought the symptoms would resolve on their own, contributing to the delay in seeking care. This interpretation aligns with findings from a study conducted in Egypt, where patients demonstrated a negative outlook on cancer symptoms, including a lack of awareness about BC, denial of having BC, and a belief that the mass would resolve spontaneously^29^. These results emphasize the significance of BC screening initiatives, highlighting their role not just in early detection but also in promoting a proactive health-seeking behavior, especially among screening-eligible women.

In this study, about 10% of participants acknowledged a delay in seeking medical attention upon recognizing a potential BC symptom, reporting at least one barrier to early presentation. Aligning with prior research^30–32^, emotional barriers were the most prevalent, with ‘disliking the visit to healthcare facilities’ emerging as the primary barrier among screening-eligible participants. Conversely, in the screening-ineligible group, ‘feeling worried about what the doctor might find’ took precedence. The emotional aspects of seeking medical care are not only individual but are also shaped by social interactions and perceptions. The patient’s social network plays a crucial role in influencing the decision to seek medical care^23^. This highlights the complex interplay between health, cultural, and social factors.

Additionally, despite the availability of a fully accessible and cost-free screening program^8^, socio-demographic and healthcare delivery barriers persist. Systemic challenges, such as limited accessibility to screening services and a lack of tailored communication strategies from healthcare professionals and families, can contribute to mistrust^33^. This reinforces the need for culturally sensitive healthcare approaches to improve engagement and trust in preventive care. Therefore, future BC campaigns should consider extending their focus to include men and promoting family involvement in the decision-making process, which may help in decreasing the emotional barriers and thus improve early seeking of medical advice. Furthermore, both screening-eligible and ineligible women identified the limited availability of female doctors in the Palestinian Ministry of Health facilities as the predominant service-related barrier. Consequently, prioritizing the increase in the number of female doctors becomes imperative to facilitate the early help-seeking behavior of women experiencing potential BC symptoms^20^. It is also important to mention that the similarities in displayed barriers between screening-eligible and screening-ineligible women as well as the lack of association between BC screening eligibility and reported number of barriers provide an opportunity to standardize future interventions aiming to mitigate those barriers.

### Future directions

Collaborative efforts among healthcare providers, policymakers, and community leaders are crucial for strengthening the healthcare system, improving accessibility, and maintaining the quality of mammography services in Palestine. Future initiatives should prioritize the implementation and evaluation of culturally sensitive educational interventions and community outreach programs to enhance BC awareness and address barriers to early symptom presentation. Emphasis should be placed on communicating the significance of non-lump breast changes, highlighting the potential curability of BC when detected early, and fostering a positive perception of healthcare providers and facilities in Palestine. Extending campaigns to involve men and families in decision-making processes may further alleviate emotional barriers and enhance participation in mammography screening. Finally, efforts should be made to increase the number of female healthcare workers in order to enhance women’s comfort when receiving treatment for BC or even while undergoing screening mammography.

### Strengths and limitations

The major strengths of the study are the large sample size, high response rate, and broad geographical coverage. However, there are some limitations. The use of convenience sampling may limit the generalizability of the findings. Nevertheless, the large participant pool and representation from diverse geographical regions may have mitigated this limitation. The exclusion of individuals with medical backgrounds and those attending oncology departments may potentially have reduced the representation of participants presumed to have certain health-seeking behavior. Moreover, there could be a potential for recall and social desirability bias in self-reported data. Nonetheless, our questionnaire used neutral, non-leading questions to minimize response bias. Additionally, data collectors were trained to maintain consistency in administering surveys.

Lastly, participants’ anticipated time and perceived barriers to seeking BC care were assessed, which may differ from evaluating those aspects among patients diagnosed with the disease. We acknowledge that our cross-sectional design does not assess whether participants actually sought medical care after experiencing symptoms. To address this, future research should incorporate qualitative methods, such as focus groups or in-depth interviews, to gain deeper insights into personal and cultural barriers. Additionally, longitudinal studies using a probability sampling approach would be valuable for tracking participants’ health-seeking behaviors over time, providing a clearer understanding of their decision-making processes and long-term engagement with screening programs.

## Conclusions

The inclination to seek immediate medical advice for various BC symptoms differed among screening-eligible and ineligible groups. Emotional barriers were consistently reported as the most prevalent barriers in both groups. Notably, there was no observed association between eligibility for BC screening and the total number of reported barriers. Similarly, no significant associations were identified between BC screening eligibility and the reported number of each barrier type, including emotional, service-related, or practical barriers. To address these barriers, culturally tailored educational interventions are essential and should align with the specific needs and beliefs of the Palestinian population.

## Data Availability

The datasets used and analyzed during the current study are available from the corresponding author on reasonable request.
